# Extracellular vesicle-derived TP53BP1, CD34, and PBX1 from human peripheral blood serve as potential biomarkers for the assessment and prediction of vascular aging

**DOI:** 10.1186/s41065-023-00306-8

**Published:** 2024-01-03

**Authors:** Yichao Wen, Haiyang Chen, Yu Wang, Yiqing Sun, Fangfang Dou, Xiling Du, Te Liu, Chuan Chen

**Affiliations:** 1 Shanghai Geriatric Institute of Chinese Medicine, Shanghai University of Traditional Chinese Medicine, 365 South Xiangyang Road, Shanghai, 200031 China; 2 Department of Neurology, Shanghai General Hospital, Shanghai Jiao Tong University School of Medicine, Shanghai, 200080 China; 3 Eberly College of Science, Penn State University, University Park, PA USA; 4 School of Life Science and Technology, Tongji University, Shanghai, 200092 China

**Keywords:** Vascular senescence, Extracellular vesicles (EVs), Biomarkers

## Abstract

**Background:**

Vascular aging is an important pathophysiological basis for the senescence of various organs and systems in the human body, and it is a common pathogenetic trigger for many chronic diseases in the elderly.

**Methods:**

The extracellular vesicles (EVs) from young and aged umbilical vein endothelial cells were isolated and identified by qPCR the differential expression levels of 47 mRNAs of genes closely related to aging in the two groups.

**Results:**

There were significant differences in the expression levels of 18 genes (we noted upregulation in PLA2G12A, TP53BP1, CD144, PDE11A, FPGT, SERPINB4, POLD1, and PPFIBP2 and downregulation in ATP2C2, ROBO2, RRM2, GUCY1B1, NAT1-14, VEGFR2, WTAPP1, CD146, DMC1, and GRIK2). Subsequent qPCR identification of the above-mentioned genes in PBMCs and plasma-EVs from the various age groups revealed that the trend in expression levels in peripheral blood plasma-EVs of the different age groups was approximately the same as that in PBMCs. Of these mRNAs, the expression of four genes–PLA2G12A, TP53BP1, OPRL1, and KIAA0895–was commensurate with increasing age. In contradistinction, the expression trend of four genes (CREG1, PBX1, CD34, and SLIT2) was inversely proportional to the increase in age. Finally, by taking their intersection, we determined that the expression of TP53BP1 was upregulated with increasing human age and that CD34 and PBX1 were downregulated with increasing age.

**Conclusion:**

Our study indicates that human peripheral blood plasma-EV-derived TP53BP1, CD34, and PBX1 potentially comprise a noninvasive biomarker for assessing and predicting vascular aging.

## Introduction

The process by which an organism’s ability to adapt to its environment is progressively reduced and where it gradually tends to die is known as senescence. The onset of senescence thus involves a decline in an organism’s ability to adapt to its environment, eventually resulting in its death [1, 2, 4, 5, 12, 15, 20] The classical features of aging include genomic instability, telomere depletion, cellular senescence, loss of protein homeostasis, dysregulated nutrient sensing, mitochondrial dysfunction, stem cell depletion, impaired intercellular communication, epigenetic alterations, and the development of a new generation of cells [1, 2, 4, 5, 12, 15, 20]. If a specific senescence biomarker system could be established based on the above characteristics of senescence, then it might facilitate the tracking and identification of senescence and its follow-up assessment [1, 2, 4, 5, 12, 15, 20] The cardiovascular system in mammals is one of the largest in the animal kingdom, and the cardiovascular system (as a large mammalian organ) is particularly important for maintaining the normal physiological functions of an organism [1, 14, 15, 27] It is essential for the health of an organism, and the health of the organism is dependent upon the proper functioning of the cardiovascular system. As an animal is subjected to continuous mechanical and metabolic stresses during the aging process, there occurs progressively impaired functioning at the molecular, cellular, and organ levels, thereby increasing the risks of disease and death [1, 14, 15, 27].Vascular aging leads to decreased vascular elasticity and abnormal lipid deposition, resulting in plaque formation and ultimately leading to aging-related diseases such as atherosclerosis, coronary heart disease, and cerebral infarction [1, 14, 15, 27]. Due to the highly heterogeneous nature of vascular walls in cells from different sources, the cellular composition and molecular characteristics of aged blood vessels in humans remain unclear; and this then limits to a degree our in-depth understanding of the vascular aging mechanism and constrains the prevention and treatment of cardiovascular diseases. Therefore, if a set of cardiovascular biomarkers with high sensitivity, specificity, rapidity, and convenience can be uncovered, it would greatly facilitate real-time and precise detection of cardiovascular aging. Kroemer et al. suggest that impaired macroautophagy, loss of protein homeostasis, genomic instability (particularly clonal hematopoiesis of uncertain potential), epigenetic alterations, mitochondrial dysfunction, cellular senescence, dysregulation of neurohormonal signaling, and inflammation are critical contributors to cardiovascular aging [1, 31]. However, the aforementioned factors also cause aging in other organs and are not truly specific indicators of cardiovascular aging. Zhang et al. demonstrated that chemokine CCL17 knockdown reduced the expression of vascular fibrosis, elastin fiber breakage, senescence marker p21, and senescence-associated secretory phenotype biomarkers in senescence-induced mice and that CCL17 also attenuated Ang II-induced vascular arteriolar stiffness, systolic-diastolic dysfunction, pathologic vascular remodeling, fibrosis, and the senescence-associated secretory phenotype. Thus, a concept of CCL17 as a novel target in the treatment of cardiovascular aging has now been proposed [31]. Qu et al.’s work revealed that transcription factor FOXO3A reflected a key molecular node in the network of differentially expressed genes (DEGs) that regulate arteriolar vasculature and that FOXO3A expression was downregulated in all six types of senescent vascular wall cells, an important feature of primate arteriolar vasculature senescence [29]. Wu et al. showed that the cyclic RNA circGNAQ regulated vascular endothelial cell senescence and delayed atherosclerosis through the circGNAQ/miR-146a-5p/PLK2-signaling pathway [28]. In addition, Zhang et al. reported that SIRT2 regulated mitochondrial oxidative stress in vascular cells, remodeled the vascular cell transcriptome, and inhibited activation of vascular senescence by inducing a de-acetylation modification of the lysine 81 site of the p66Shc protein. Thus, these authors demonstrated for the first time that SIRT2, as an epigenetic regulator, regulated vascular senescence through a cytoplasmic-mitochondrial shuttle mechanism [30]. All of the above-mentioned studies therefore provide a foundation for the screening of cardiovascular senescence markers.

Extracellular vesicles (EVs) are formed by intracellular endosomes released into the extracellular environment by exocytosis, and they range in diameter from 20 to 200 nm. EVs are phospholipid bilayer vesicles secreted by almost all types of cells and are found in various bodily fluids such as blood, urine, cerebrospinal fluid, saliva, and ascites [27] Based on their production process and diameter, EVs are categorized as exosomes, microvesicles (MPs), and apoptotic bodies. EVs can carry functional proteins, nucleic acids, lipids, and other biologically active molecules that are delivered to recipient cells and mediate physiologic and pathologic processes in the cells, including inflammation, autoimmune responses, endothelial dysfunction/injury, procoagulation, angiogenesis, endothelial proliferation, and other vasculitis-related pathologic conditions [27]. EVs of different cellular origins have also been shown to be associated with vasculitis. In addition, EVs from different cellular sources serve different functions, e.g., endothelial cell-derived EVs are primarily involved in vascular inflammation, injury, and angiogenesis; while platelet-derived EVs may be involved in procoagulant reactions [27]. EVs also play a pro-angiogenic role via the expression of VEGF, bFGF, PDGF, MMPs, miR-125a, and miR-31 or inhibit angiogenesis by increasing the production of reactive oxygen species (ROS) and downregulating VEGFR2 and pERK1/2 protein levels [27]. Furthermore, EVs (as carriers of intracellular substances) are abundant in peripheral blood and are easily separated and enriched. EVs thereby constitute a potential and sensitive source of noninvasive diagnostic markers.

Song et al. recently applied a multi-omics technique to obtain 47 genes that were closely related to cellular senescence [24] and thus likely to be potential predictors of cellular senescence. We thus envision that this study will provide us with novel concepts and mechanisms underlying vascular aging. We herein collected peripheral blood from young to old individuals, with one age group encompassing 10 years; isolated and enriched EVs; and attempted to uncover biomarkers associated with vascular aging using qPCR.

## Materials and methods

### Collection of human peripheral blood samples

Our healthy individual cohort consisted of 56 people, with eight in each age group– four males and four females (from 10 to 70 years of age, with every 10th individual constituting an age group). The aforementioned individuals were in good health, with none showing any disease; and all physical examination indicators were within the normal range. Five milliliters of peripheral blood was then collected from each individual. The study involving human peripheral blood has been obtained according to consent regulation and approved by the Ethics Review Committee of Shanghai University of Traditional Chinese Medicine in Human Production authorized by Shanghai Municipal Government (No. 2018LCSY035); meanwhile, the informed consent has been provided by all patients conducted in accordance with the Declaration of Helsinki.

### Human umbilical vein endothelial cell (HUVEC) isolation and culture

The umbilical cords from healthy puerperal women were collected aseptically after parturition and isolated by treatment with 1% trypsin as previously described [14]. The HUVECs were grown on 1% gelatin-coated culture plates in McCoy’s 5 A medium (Sigma-Aldrich, St. Louis, MO, USA) supplemented with 15% fetal bovine serum (HyClone), 100 U/ml penicillin, and 100 µg/ml streptomycin (HyClone) at 37 °C in a humidified atmosphere containing 5% CO_2_ in compressed air. The cells were used within two passages.

### Collection the EVs from HUVECs and human PBMCs

EVs were isolated from human peripheral blood plasma according to a previously established ultracentrifugation method [9, 13]. Briefly, human peripheral blood plasma was collected and centrifuged at 500xg for 10 min to remove cellular fragments and apoptotic bodies, and the supernatant was then centrifuged at 16,500xg for 20 min to collect MPs. The supernatant was passed through a 0.22-µm filter (Merck Millipore Ltd, Tullagreen, Carrigtwohill, Co. Cork, Ireland) to remove proteins and fragments, and the EVs were ultimately precipitated by ultracentrifugation at 118,000xg for 70 min and resuspended in saline.

### Electron microscopic analysis

The EVs were diluted to 1 mg/mL with PBS and placed onto a glow-discharge copper grid on a filter paper and dried under an infrared lamp for 20 min. Then, 3% (w/v) phosphotungstic acid (Electron Microscopy Sciences, Washington, PA, USA) was added and the grid was air-dried at room temperature. We examined the exosomes under a transmission electron microscope with an accelerating voltage of 80 kV (Hi-Rel 600; Hitachi, Tokyo, Japan).

### qPCR

Total RNA was extracted from each group of cells with the TRIzol Reagent (Invitrogen, USA) according to the instructions. Total RNA was treated with DNase I (Sigma-Aldrich, USA), quantified, and reverse-transcribed to cDNA using ReverTra Ace-α First-Strand cDNA Synthesis Kit (TOYOBO). qRT-PCR was conducted in a RealPlex4 real-time PCR detection system (Eppendorf Co., Ltd., Germany), and the cDNA was analyzed using a SyBR Green RealTime PCR Master MIX (TOYOBO), and a fluorescent dye was used for nucleic acid amplification. The qRT-PCR involved a total of 40 amplification cycles: denaturation at 95 °C for 15 s, annealing at 58 °C for 30 s, and primer template extension at 72 °C for 42 s. Relative gene expression was determined using the 2^−ΔΔCt^ calculation method: ΔCt = Ct_genes – Ct_18s RNA; ΔΔCt = ΔCt_all_groups – ΔCt_blank control_group. The mRNA expression levels were corrected for the expression levels of 18s rRNA.

### Western blot analysis

Briefly, the total proteins of each group of cells were used for 12% SDS-PAGE denaturing gel electrophoresis, and after completion, they were transferred to PVDF membranes (Millipore). After sealing and washing the membranes, we performed incubation with primary antibodies for 45 min at 37 °C. After sufficient membrane washing, incubation with secondary antibodies was performed for 45 min at 37 °C. The membranes were washed four times with TBST for 14 min each at room temperature and exposed to allow development (Sigma-Aldrich Chemical) with ECL-enhanced chemiluminescence (ECL Kit, Pierce Biotechnology).

### Statistical analysis

Each experiment was performed as least three times, and data are shown as the mean and standard error, where applicable; differences were evaluated with Student’s *t* test, with a *P* value less than 0.05 considered to be statistically significant.

## Results

### Young vs. old HUVECs-EVs contain DEGs mRNAs

**Fig. 1:**
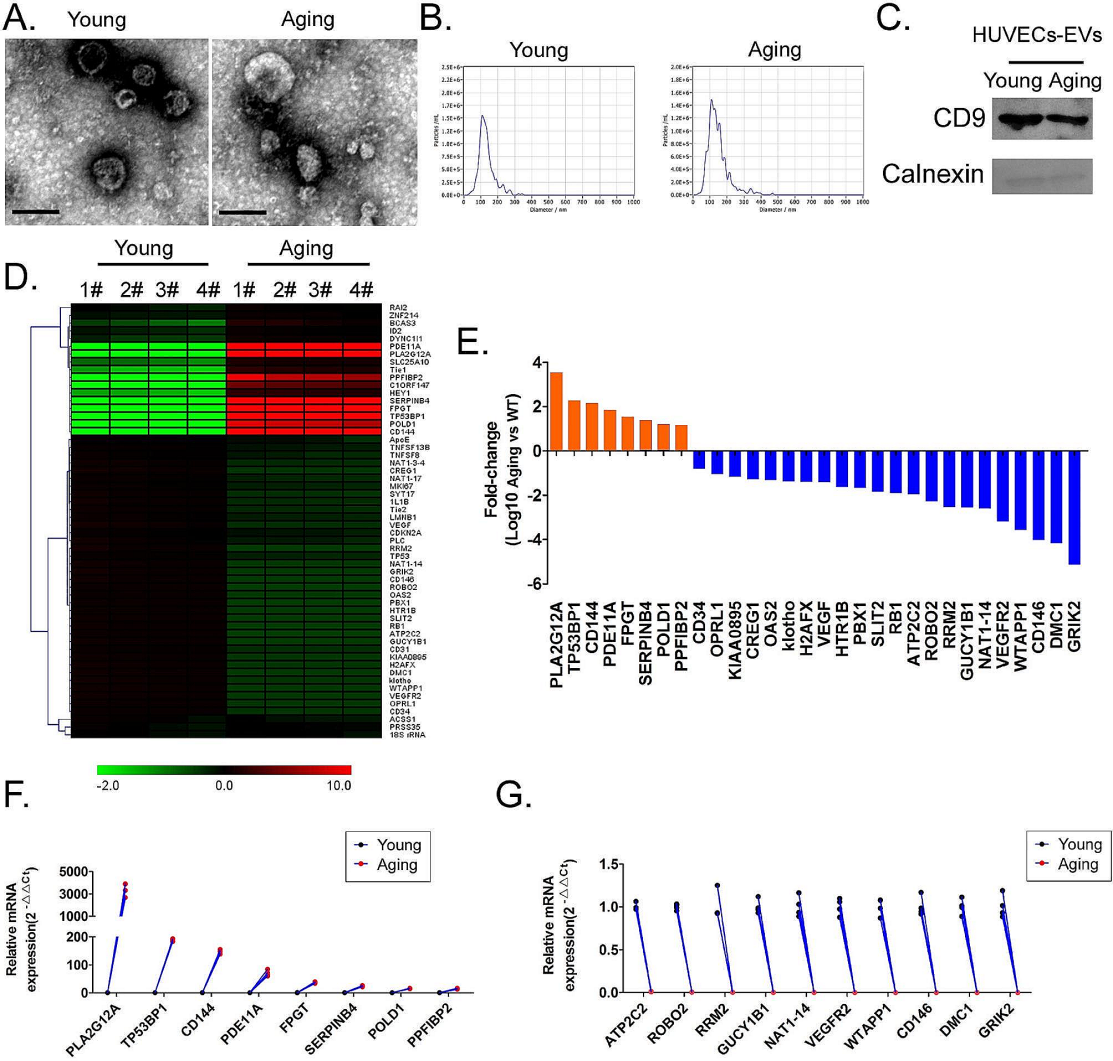
Differential analysis of the expression levels of senescence-related genes in EVs of HUVEC origin. (**A**) Transmission electron microscopic evaluation of EV phenotype (Scale bar = 200 nm). (**B**) Particle size analysis of EVs. (**C**) Western blot results of EV markers. (**D**) Heatmap analysis of qPCR assay results of gene-expression levels. (**E**) Analysis of genes with significant expression differences. (**F**) qPCR results of gene expression showing a positive correlation with the senescence process. (**E**) qPCR results of gene expression showing a negative correlation with the senescence process

By means of successive passaging, we selected the sixth generation of HUVECs as the senescent group (Aging) and the second-generation HUVECs as the control group (Young). The cell-derived EVs from each group were collected by ultracentrifugation. Both transmission electron microscopy and particle sizing assays confirmed that the EVs exhibited typical characteristics of exosomal morphology, with diameters between 10 and 200 nm (Fig. 1A and B). Western blotting results indicated that the cell-derived EVs from each group expressed the exosomal markers CD9 but did not express the cytoplasmic protein calnexin (Fig. 1C). We subsequently examined the expression levels of 47 genes closely correlated with cellular senescence as well as vascular endothelial (progenitor) cell markers such as Tie1, Tie2, CD31, CD146, VEGFR2, VEGF, PLC, CD144, and CD34 in the EVs of each group of samples using qPCR (Fig. 1D). The qPCR results indicated that the expression levels of eight genes were significantly higher (fold-change [Aged/Young] > 10) and those of 22 genes were significantly lower (fold-change [Age/Young] < 0.1, Fig. 1E) in the aged HUVECs-EVs compared to the control group. Considering the wide range of genes with downregulated expression and by adjusting the threshold range (fold-change [Age/Young] < 0.01), we finally selected 18 genes with the most significant differences in expression levels (the significantly upregulated genes were PLA2G12A, TP53BP1, CD144, PDE11A, FPGT, SERPINB4 POLD1, and PPFIBP2; and the significantly downregulated genes were ATP2C2, ROBO2, RRM2, GUCY1B1, NAT1-14, VEGFR2, WTAPP1, CD146, DMC1, and GRIK2) and used them in subsequent studies (Fig. 1F and G). Our results revealed significant differences in gene expression levels in EVs released from the aged and young HUVECs.

### Significant differences in the expression levels of senescence-related genes in leukocytes from different age groups

**Fig. 2:**
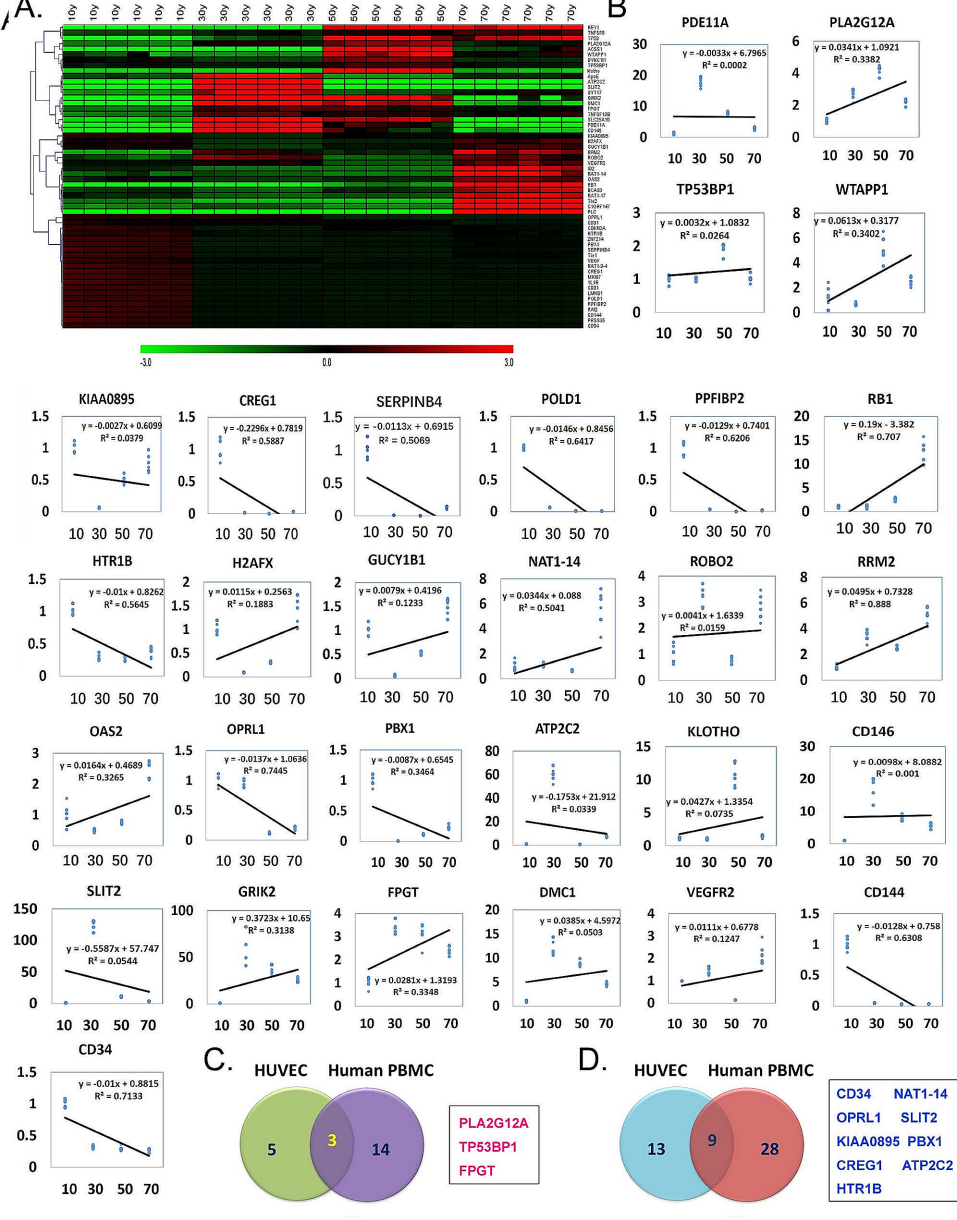
Differential analysis of the expression levels of senescence-related genes in PBMCs from individuals of various ages. (**A**) Heatmap analysis of qPCR assay results for gene-expression levels. (**B**) Analysis of the correlation between the expression level of each gene and increase in age. (**C**) Results of the intersections between HUVECs-EVs and highly expressed genes of PBMC origin. (**D**) Results of the intersections between HUVECs-EVs and low-expressed genes of PBMC origin

Many previous reports have indicated that the gene-expression profiles of human PBMCs are closely associated with human organ aging [17, 32]. There was a certain pattern in the gene expression levels of PBMCs in the composition of aging in the body, and there was a certain relationship between them and aging inheritance. Therefore, in this study, we determined the expression levels of senescence-related genes in PBMCs from different age groups were determined [17, 32]. We collected PBMCs from individuals of all ages and examined the expression levels of 47 genes associated with aging and markers of vascular endothelial (progenitor) cells using qPCR. qPCR results showed that some of the aforementioned genes were significantly differentially expressed in PBMCs from individuals of different ages (Fig. 2A). Among these genes, the expression levels of KIAA0895, H2AFX, GUCY1B1, RRM2, ROBO2, VEGFR2, ID2, NAT1-14, OAS2, RB1, BCAS3, NAT1-17, Tie2, C1ORF147, and PLC were lower in the young adult population (10–50 years of age), and the expression levels of above genes were significant in the elderly population (70 years of age) showed significantly higher expression levels (Fig. 2A and B). The expression levels of genes such as PLA2G12A, WTAPP1, RB1, H2AFX, GUCY1B1, NAT1-14, RRM2, GRIK2, FPGT, OAS2, DMC1, and TP53BP1 were upregulated with increasing age (Fig. 2B), and the expression levels of genes such as CREG1, SERPINB4, POLD1, PPFIBP2, HTR1B, OPRL1, PBX1, SLIT2, CD144, and CD34 showed were downregulated with increasing age (Fig. 2B). We subsequently intersected the gene-expression levels in PBMCs from individuals of different ages with those from HUVEC-EV sources, and the results revealed that both exhibited upregulation of the DEGs such as PLA2G12A, TP53BP1, FPGT, and three others (Fig. 2C); while both showed downregulation of CD34, OPRL1, KIAA0895, HTR1B, PBX1, SLIT2, ATP2C2, NAT1-14, CREG1, and nine other genes (Fig. 2D).

### Significant differences in the expression levels of age-related genes in peripheral blood plasma EVs of different age groups

**Fig. 3:**
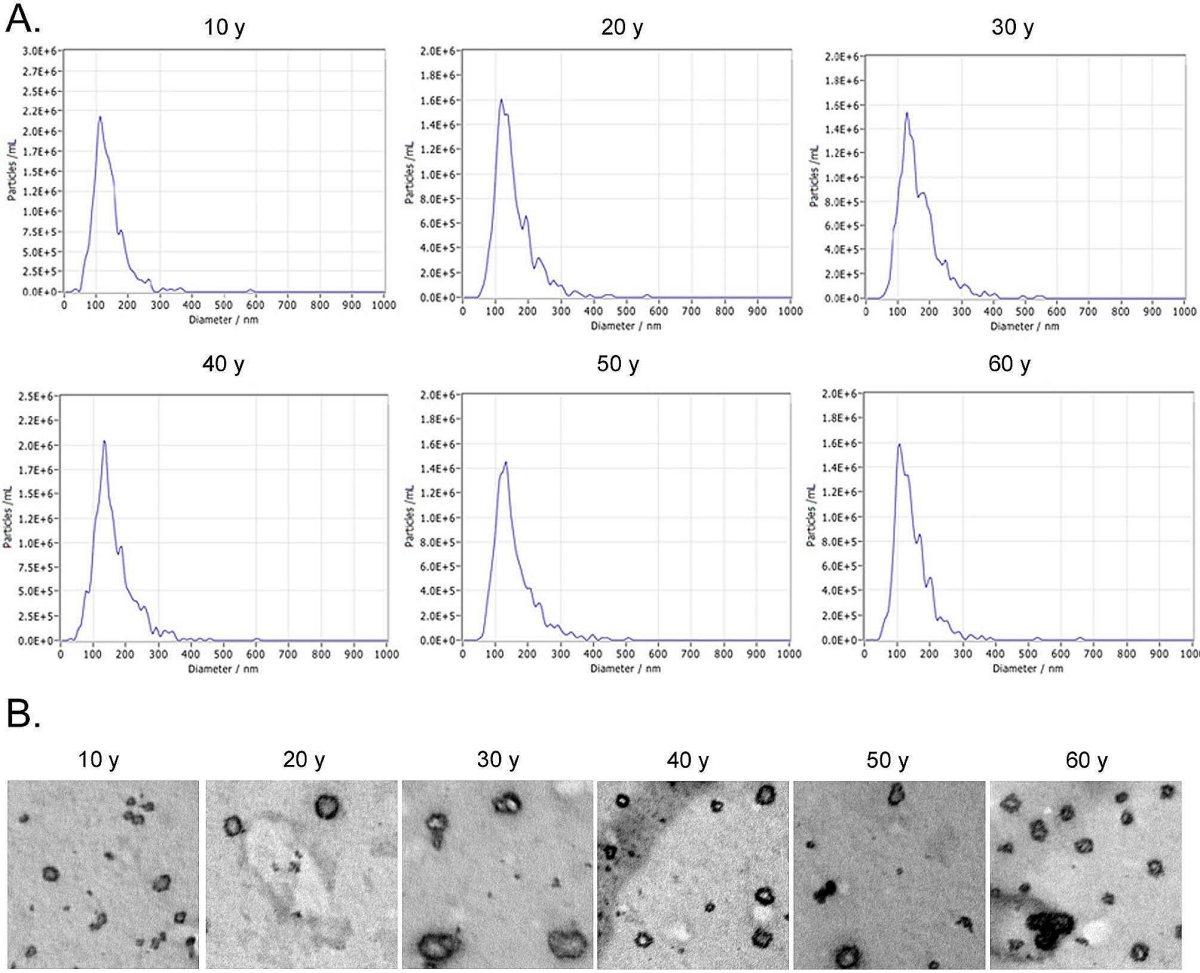
Structural morphology and particle size analysis of human peripheral blood plasma EVs. (**A**) Particle size analysis of EVs. (**B**) Transmission electron microscopic evaluation of EV phenotype (Scale bar = 200 nm)

**Fig. 4:**
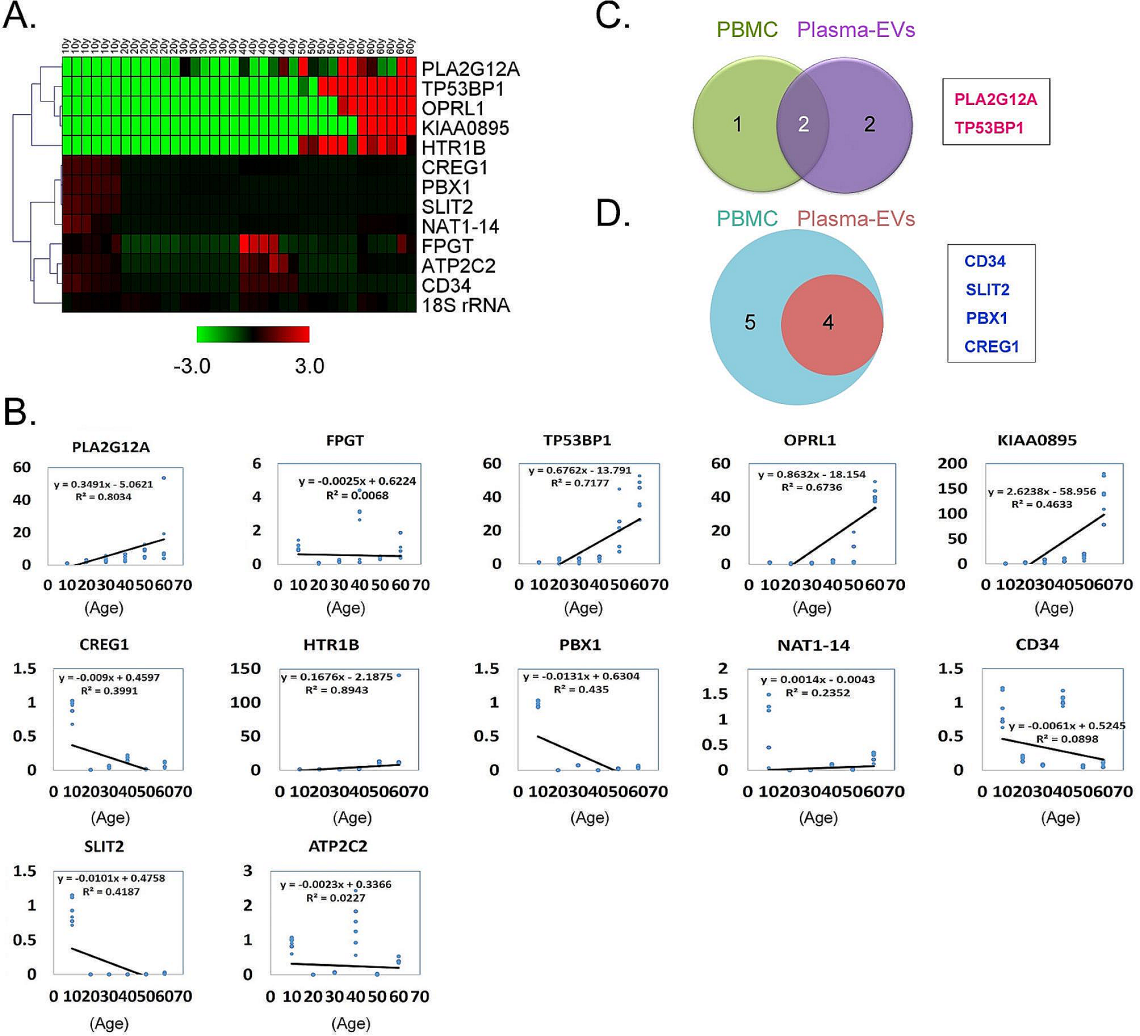
Differential analysis of the expression levels of senescence-related genes in human peripheral blood plasma EVs. (**A**) Heatmap analysis of qPCR results of the expression levels of each gene in plasma EVs. (**B**) Analysis of the correlation between the expression level of each gene in plasma EVs and increase in age. (**C**) Results of the intersections between highly expressed genes of PBMC origin with plasma-EVs. (**D**) Results of the intersections between low-expressed genes of PBMC origin and plasma-EVs

To determine whether the expression levels of the above-mentioned nine genes in peripheral blood plasma EVs from various age groups also correlated with aging, we collected peripheral blood plasma from the groups and isolated their EVs. Using transmission electron microscopy and particle sizing assays, we confirmed that EVs from human plasma sources–as well as those from the supernatants of HUVEC culture medium–had typical exosomal morphology characterized by diameters between 10 and 200 nm (Fig. 3A and B). The qPCR results showed that the expression level trends for the aforementioned nine genes in peripheral blood plasma EVs from the different age groups were approximately the same as for those in leukocytes (Fig. 4A). Among these genes, the expression trends for four genes (PLA2G12A, TP53BP1, OPRL1, and KIAA0895) were proportional to the increase in age (Fig. 4B); in contrast, the expression trends for four genes (CREG1, PBX1, CD34, and SLIT2) were inversely proportional to increasing age (Fig. 4B). We then intersected 12 DEGs screened from PBMCs from individuals of all ages with eight genes derived from plasma EVs, and the data revealed that the genes upregulated in both were PLA2G12A, TP53BP1, and two others (Fig. 4C); while the four genes whose expression was downregulated in both were CD34, PBX1, SLIT2, and CREG1 (Fig. 4D).

## Discussion

Organ function declines as the body ages, and the risk of various diseases such as cardiovascular disease, cancer, and neurodegenerative diseases increases [16]. The accumulation of senescent cells in the aging body promotes the development of age-related diseases, and cellular senescence occurs throughout the life of an individual organism and plays an important role in various physiologic as well as pathologic processes [16]. Therapeutic measures that target senescent cells through selective elimination or modulation of senescence-associated secretory phenotype (SASP) have been reported to ameliorate a variety of age-associated pathologic processes and prolong lifespan in mice [16]. Functional decline brought about by organ aging (usually manifested clinically as a variety of aging-related diseases) seriously affects the health and quality of life of the aging population. Scientists have increasingly explored the aging markers of multiple organs/systems (e.g., brain, heart, blood vessels, lungs, skeletal muscle, liver, kidney, bone, fat, skin, intestines, pancreas, reproductive system, hematopoietic system, and immune system) from the perspectives of physiologic, imaging, and histologic features; cellular and molecular changes; and humoral-secretion factors–providing an important reference for scientifically assessing the degree of organ aging and thus generating early warnings of aging-associated degenerative diseases [2, 5, 12] Biomarkers of aging therefore constitute the most important indicators of organ senescence. Aging biomarkers are physiologic and molecular indicators of age-related structural or functional degeneration at the organismal, organ, tissue, cellular, and subcellular levels; and these can then be used to monitor and assess the biological changes that accompany aging and thereby predict the degree of the evolution of organ senescence with respect to pathology. This information will facilitate understanding the relationships and discrepancies between ages of the various tissues or organs of the body and their actual chronological age. The establishment of screening biomarkers of aging contributes to the development of appropriate aging assessments and early-warning indices, as well as intervention in aging-related diseases [2, 5, 12]. López-Otín et al. were the first to propose 12 markers of aging that included (1) the basic aging markers (accumulated over time, with clear accelerating effects on aging) of genomic instability, telomere attrition, epigenetic alterations, loss of proteostasis, and disabled macroautophagy; (2) antagonistic markers of senescence (with opposite effects on senescence to varying degrees) that entail deregulated nutrient-sensing, mitochondrial dysfunction, and cellular senescence; and (3) senescence composite markers (appearing when the organism’s aging damage cannot be repaired) such as stem cell exhaustion, altered intercellular communication, and dysbiosis [15] These authors described additional three markers of loss of macroautophagy, chronic inflammation, and ecological dysregulation [15]. The above findings are of great significance in understanding the onset and progression of aging and how to intervene in the aging process. Keller et al. mapped whole-body ncRNA expression in aged and rejuvenated mice and identified a set of broadly dysregulated microRNAs that may act as systemic regulators of aging via plasma and EVs [25]. This study suggests an intrinsic link between senescence-related factors and EVs. All of these studies indicate the value and usefulness of aging markers in evaluating, measuring, and predicting aging.

As one of the most important organs within an animal, blood vessels maintain the normal physiologic and biochemical functions of the organism. Vascular aging, then, constitutes a major pathophysiological basis for the aging of various organs and systems in the human body, and it reflects a common pathogenesis of numerous chronic diseases in the elderly [22]. Along with chronological aging, the incidence rates of diseases related to vascular aging (including atherosclerosis, hypertension, and cardiovascular and cerebrovascular diseases) have been increasing in the elderly population, posing a serious risk to human health [22]. The chief mechanisms of vascular aging include vascular cellular senescence, inflammation, oxidative stress, endothelial dysfunction, reduced telomerase activity, lipid metabolism disorders, mitochondrial dysfunction, and dysregulation of the intestinal flora; and the interplay between and among these factors has generated complex underlying pathological mechanisms [22]. Given that the aging of the cardiovascular system exerts a huge impact on the normal functioning of the body, it is particularly crucial to establish an effective mechanism for monitoring and assessing aging. Although a plethora of studies have reported some close links between the expression levels of genes or proteins and cardiovascular aging, there is still no systematically established or reported noninvasive, sensitive, and simple biomarker for measuring, assessing, and monitoring the degree of vascular aging in the body using peripheral blood EV contents. In the present study, we were inspired by the report of Song et al. [24], and we attempted to uncover gene clusters in peripheral blood EVs from people of all ages that were consistent with the expression levels (i.e., trends) of genes associated with vascular endothelial aging.

After our step-by-step testing, we narrowed the candidate biomarkers to three genes, i.e., plasma EV-derived TP53BP1, CD34, and PBX1. TP53BP1 expression was upregulated with increasing age, and CD34 and PBX1 expression was downregulated with increasing age. TP53BP1 is the P53-binding protein 1 (53BP1), i.e., a p53-binding mate that enhances p53 transcriptional activity [10, 11] TP53BP1 consists of two BRCA1 carboxyl-terminal (BRCT) structural domains that bind to p53 and a separate structural domain responsible for binding to phosphorylated histone H2A.X [18]. It has been reported that when human cells age or undergo mitotic arrest, phosphorylation modification of TP53BP1 occurs at Ser1618 [6]. These results suggest a positive correlation between TP53BP1 activation and cellular aging and DNA damage. CD34 is a type I transmembrane glycosylated phosphoprotein expressed by hematopoietic stem/progenitor cells, vascular endothelium, and some fibroblasts [8] and is a recognized marker of hematopoietic stem cells. CD34 is highly expressed when hematopoietic stem (progenitor) cells are naïve in “stemness” and is completely lost when hematopoietic stem (progenitor) cells differentiate into mature lymphopoietic-lineage cells or when they age [3, 7, 21, 23]. Pre-B-cell leukemia transcription factor 1 (Pbx1) is a member of the TALE family [19]. Pbx1 mRNA produces two transcripts, Pbx1a and 1b, during variable splicing; and Pbx1 is commonly expressed in embryonic and adult tissues [19]. Intriguingly, it was shown that enhanced expression of a Pbx1-Sirt1 positive-feedback loop significantly attenuated ROS-induced senescence and apoptosis in hair follicle-derived mesenchymal stem cells [26], suggesting that Pbx1 expression was negatively correlated with senescence. The above reports are in close agreement with our experimental results and indirectly support our findings and scientific hypotheses.

## Conclusions

In summary, our study indicates that human peripheral blood EVs derived from TP53BP1, CD34, and PBX1 constitute a noninvasive, provisional biomarker for assessing and predicting vascular aging.

## Data Availability

The datasets used or analysed during the current study are available from the corresponding author on reasonable request.

## Abbreviations

EVs: Extracellular vesicles
DEGs: Differentially expressed genes
HUVEC: Human umbilical vein endothelial cell
qPCR: Quantitative real-time PCR
H&E: Hematoxylin-eosin staining
